# Skin phenotype diversity across populations: from skin morphology to molecular phenotype

**DOI:** 10.3389/fmed.2026.1721899

**Published:** 2026-08-27

**Authors:** Xiaoqian Yu, Meiting Yi, Jiayuan Shi, Yan Jia, Liya Song, Huaming He

**Affiliations:** 1 School of Light Industry Science and Engineering, Beijing Technology and Business University, Beijing, China; 2 Beijing Technology and Business University, College of Light Industry Science and Engineering, Academy for Interdisciplinary Studies, Beijing, China

**Keywords:** lipid, microecology, molecular phenotype, physiological indicators, skin

## Abstract

The considerable range of phenotypic variation observed in human populations may partly reflect distinct processes of natural selection and adaptation to variable environmental conditions. Differences in physiological indices, structure, molecular phenotypes and metabolic mechanisms of the skin across populations with varying skin phototypes are also a reflection of this unique process. These differences are reflected in skin color, pH, moisture content, water loss, sebum secretion, and skin elasticity. The differences in skin properties are determined by variations in molecular phenotypes, such as morphology, gene expression, protein composition, lipid metabolism, and skin microecology. These molecular phenotypes, which are influenced by a complex interplay of genetic, environmental, and social factors, cluster differently across populations that are often categorized by race or ethnicity in the cited literature. Understanding skin morphology and molecular phenotypes across populations with diverse skin phototypes will allow for a more comprehensive understanding of the molecular mechanisms underlying differences in skin properties, and for more accurate dermatological diagnosis, skin management, and regulation of related products.

## 1 Introduction

The substantial phenotypic variation observed across human populations may partially reflect distinct processes of natural selection and adaptation to variable environmental conditions (1). Natural selection has shaped regional adaptations in modern human groups by influencing morphological and disease-related diversity, with pronounced manifestations in cutaneous pigmentation, pilosebaceous development, and immune response pathways (1, 2). Populations are typically classified into groups based on genetic clustering patterns. These groups exhibit functional conservatism in skin characteristics while displaying differences in structural, functional, and molecular phenotypic traits (3–8). Such variations not only govern cutaneous morphology but also determine differential responses to environmental insults, disease susceptibility profiles, and therapeutic requirements. Population-specific disparities in architecture, genetic loci, inflammatory signaling cascades, and microbiome configurations have evolved through natural selection as adaptive responses to localized ecological pressures over evolutionary timescales (9). For instance, the high melanin content in equatorial populations demonstrates positive selection for UVR protection, while reduced ceramide synthase activity in certain groups correlates with arid climate adaptations. This evolutionary framework underscores the necessity of integrating population genetics into dermatological research to decode ethnic-specific pathomechanisms and optimize targeted interventions.

In this review, we use the population descriptors (e.g., Black, White, East Asian, Indian) reported in the primary studies we cite. We recognize that these categories are social and political constructs rather than fixed biological determinants. They reflect self-identified or investigator-assigned groupings in the original literature, and we employ them here solely as a heuristic framework to synthesize and compare findings. Wherever possible, we emphasize the underlying skin phenotypes—such as melanin content, barrier function, and lipid composition—as the primary analytical variables. The skin consists of the epidermis and the dermis. Keratinogenic cells in the epidermis differentiate to produce keratin, which forms the protective stratum corneum and constitutes the first physical barrier of the skin, while the dermis is rich in extracellular matrix proteins such as collagen and elastin, which are synthesized and maintained by fibroblasts to give the skin elasticity and strength (6, 10, 11). In addition, accessory organs such as hair follicles, sweat glands, and sebaceous glands play key roles in regulating body temperature and sebum secretion. Skin appearance characteristics include color, texture and texture. Color is determined by melanin, texture is related to stratum corneum metabolism, water retention capacity and dermal structural integrity, and texture is influenced by genetic, age and environmental factors. At the molecular level, skin health is regulated by a variety of molecules; loss of lipids and proteins leads to dryness, reduced elasticity, and wrinkle formation, insufficient water and natural moisturizing factors lead to roughness and flakiness, and a reduction in antioxidant molecules accelerates skin aging (12, 13). There are significant differences in these molecular phenotypes in skin of different populations, which directly affect the skin’s response to environmental stimuli and care needs.

Specifically, the skin of individuals from four population groups - White, Indian, Black and East Asian - exhibits significant differences in structural and molecular phenotypes. Skin of individuals with lighter phototypes is typically lower in melanin and is more susceptible to UV damage. Individuals with darker skin phototypes tend to have a thicker stratum corneum and higher melanin content, which provides greater UV protection. East Asian skin has moderate melanin content and fine skin texture, but is susceptible to environmental factors. Indian skin, on the other hand, has unique characteristics and is usually darker in color with moderate sebum production. These racial differences not only affect the appearance and function of the skin, but also determine the strategies for skin care and disease treatment; therefore, a deeper understanding of the skin differences between different populations is important for advancing skin-related medical research, disease treatment, and cosmetic development (14–18). We will systematically summarize the variation in skin structure and molecular phenotypes as reported across studies of populations self-identified as White, Indian, Black, and East Asian.

## 2 Physiological indicators to determine skin properties

Physiological indicators of the skin are the combined result of the morphological structure and molecular phenotype of the skin. Physiological indicators of the skin are parameters used to assess skin properties such as health status and function. Common indicators are: skin color (brightness, redness, yellowness), water loss, pH, etc. These are indicators that directly reflect differences in skin condition. The differences in these indicators amongpopulations are shown in Table 1.

**Table 1:** Physiological indicators (skin color, pH, transepidermal water loss, hydration level, sebum production, skin elasticity) across population groups as reported in the cited studies (individuals identified as White, Indian, Black, East Asian).

Physiological indicators	Individuals identified as White	Individuals identified as Indian	Individuals identified as Black	Individuals identified as East Asian
Skin color	Lightest	Darker	Darkest	Lighter
pH	Lower	Higher	Higher	Higher
Transepidermal Water Loss	Highest	Higher	Lowest	Lower
Hydration Level	Lower	Higher	Highest	Lowest
Sebum production	Lower	Medium	Highest	Lower
Skin elasticity	Lower	Lower	Higher	Higher

Population descriptors (Individuals identified as White, Indian, Black, East Asian) reflect self-identified or investigator-assigned categories in the cited primary studies. These are social constructs and are used here solely to organize findings from the literature, not as biological determinants of skin properties.

### 2.1 Skin color

Skin color is the most obvious and direct feature that varies across populations. Skin color is usually measured in terms of L for lightness, a for redness, b for yellowness, and the ITA (individual type angle) calculated from Lab’s three measures; the larger the ITA, the lighter the skin color.

There are often visible differences in skin color between the populations described in the cited studies. Populations identified as Black in the cited studies tend to have the darkest skin phototypes, followed by those identified as Indian, then East Asian, and finally White, reflecting the substantial variation in melanin content across these groups (19). Individuals identified as Black in the cited studies tend to have higher a-values (redness), while b-values (yellowness) are not significantly different across groups (14).

Driven by natural selection, ultraviolet radiation due to altitude, hours of sunlight, latitude and longitude have become the main cause of differences in skin color, so that individuals with the same population descriptor also exhibit a range of skin colors. For individuals identified as East Asian, the melanin and erythema indices on the cheeks of Kunming volunteers were higher compared to those of Beijing volunteers because Kunming is located at high altitude (20). In contrast, volunteers located in countries and cities with higher dimensionality, Seoul, South Korea, Beijing and Shanghai, China, had higher skin brightness than those located in low-latitude urban countries, Jakarta, Indonesia, and Kuala Lumpur, Malaysia, and the skin of women from Vietnam and Singapore was also brighter than the skin of women from Indonesia, Indonesia (21, 22). City volunteers with higher average annual sunshine hours or higher UV intensity had significantly lower lightness, redness and ITA indices. Women in Tokyo, Japan had significantly lower lightness, redness and ITA and significantly higher yellowness and hue angle than women in Beijing, China (23); volunteers in Sendai, Japan were significantly whiter than volunteers in Harbin, Shanghai, Guangzhou, China, Seoul, Korea and Manila, Philippines (24).

### 2.2 pH

The pH of the skin is usually between 4.5 and 5.5, which is essential for maintaining skin health. A weakly acidic environment helps to strengthen the skin barrier function, inhibit the growth of harmful microorganisms, maintain a balance of beneficial flora, and support enzyme activity to promote stratum corneum repair and renewal. In addition, a proper pH reduces inflammation, maintains skin moisture, and prevents dryness and irritation (25). Therefore, maintaining a normal pH of the skin is one of the key factors in keeping the skin healthy.

Studies have shown that there are stable and significant differences in skin pH among different population groups. Some studies compared individuals identified as Black, Indian, White, and East Asian; female nursing students identified as Black and White; and women identified as Black, Caribbean Mixed-race, and White (26–28). Studies have reported lower facial skin pH in White compared to Black and Indian, which may be related to differences in barrier function, requiring a lower pH to maintain the skin barrier (26–28). However, it has also been suggested that pH differences between these populations are not significant (29). In addition, skin pH can vary among populations of the same population descriptor in different regions. Among the East Asian, Indonesian, Singaporean and Korean women have higher skin pH than Vietnamese women (22, 30). This difference may be related to factors such as sebaceous glands and sweat glands and may maintain a dynamic balance with other skin barriers (e.g., physical, immune, and microbial barriers) (31–33).

### 2.3 Transepidermal water loss

Transepidermal Water Loss (TEWL) is the amount of water lost through evaporation from the skin’s surface, and is an important indicator of the skin’s barrier function. Higher TEWL values indicate a more damaged skin barrier and increased moisture loss, which can lead to dryness and sensitivity, while lower TEWL values indicate a healthy skin barrier that is able to effectively lock in moisture and maintain hydration and elasticity of the skin. Therefore, TEWL values are useful in assessing skin barrier status and moisturizing effectiveness.

In studies of TEWL across populations with varying skin phototypes, TEWL values are generally highest in individuals identified as White, followed by those identified as Indian, East Asian, and lowest in those identified as Black (34–37). The study mainly included individuals identified as Black, East Asian, and White. Overall, this difference may be related to the fact that skin physical barrier function and regulatory factors in individuals identified as White are not dominant in any of the fourpopulations. However, there are also studies that differ from these findings (24, 27, 29, 38). Such differences may be due to the age of the volunteers themselves, their skincare habits, or the environmental climate, UV exposure, temperature and humidity of the location (39–42). Studies from the same ethnicity and different regions support this idea. Volunteers from drier regions (e.g., Harbin, China) exhibited higher TEWL values compared to those from more humid regions (e.g., Shanghai, Manila, Singapore) (22, 24, 30). Guangzhou in China has a higher UV intensity compared to Shanghai in China, Seoul in South Korea and Sendai in Japan, Shijiazhuang, Nanjing, and Guangzhou in China compared to Suwon in South Korea, so volunteers in areas of high UV intensity have higher TEWL values (24, 43).

### 2.4 Hydration level

The amount of moisture in the skin can be reflected by the Hydration Level or Skin Capacitance. Both show a positive correlation with moisture content.

More studies have concluded that skin hydration is generally higher in individuals identified as Black, intermediate in those identified as Indian and White, and lowest in those identified as East Asian (29, 38, 44–46). However, not all relevant studies maintain the same conclusions (24, 27, 28, 47). This difference may be due to more factors such as age, climate, and skin care habits (47, 48).

It has been found that populations have their own best adapted ambient temperatures, and this more comfortable ambient temperature leads to higher skin hydration and better skin condition. Subjects in Sendai, Japan had higher skin surface hydration than women in Harbin, Shanghai, and Guangzhou in China, Seoul in Republic of Korea, and Manila, Philippines (24). Shijiazhuang and Guangzhou females had significantly lower skin hydration values than females in Nanjing and Suwon in Republic of Korea (43). Skin hydration in the forehead of women in Vietnam was higher than that of Indonesia, Korea, and Singapore (22, 30). Any deviation from comfortable temperatures affects skin barrier function by irritating the skin, generating inflammatory factors, and accelerating the rate of water loss, thus affecting skin moisture content (42, 49–51).

### 2.5 Sebum production

Skin Surface Lipids are the lipid components of the skin surface, mainly composed of lipids secreted by sebaceous glands and lipids produced by the breakdown of epidermal keratinocytes, which play the roles of protection, moisturisation, antioxidant, and maintenance of the skin’s microecology (52).

Total lipogenesis rates are generally higher in study populations identified as Black compared to those identified as White, and individuals identified as Indian usually have a higher amount of sebum than those identified as East Asian (24, 34). This may be related to the higher ambient temperatures and humidity in the tropical regions where many of these study populations reside, where temperature and humidity are higher and oil production is more rapid (42). In addition, it has been reported that the sebaceous gland activity is higher among the black population (53–55). The rate of sebaceous gland secretion in the same ethnic group is also similar to the pattern of different ethnic groups, with lower latitudes having higher temperatures and humidity, and therefore higher sebum secretion, e.g., the level of sebum secretion in women in Guangzhou and Nanjing is significantly higher than that in Suwon, Beijing and Shijiazhuang (43, 56).

### 2.6 Skin elasticity

Skin elasticity is an important reflection of the mechanical properties of the skin, and its level is generally closely related to the number and arrangement of collagen and elastic fibers in the skin.

Between different populations, skin elasticity is typically higher in East Asian compared to Indian, and elastic recovery is higher in Black compared to White (21, 24, 45). This may be related to the skin structure of different populations. Differences in skin elasticity among individuals with the same population descriptor are related to photoaging caused by long-term UV exposure (21). Female skin from Guangzhou, China had lower resilience fatigue values compared to Seoul, Korea, Sendai, Japan, Shanghai, China and Manila, Philippines (24). Skin elasticity of volunteers in Seoul, Korea, Beijing, China and Shanghai was better than in Ho Chi Minh City, Vietnam, Jakarta, Indonesia and Kuala Lumpur, Malaysia (21). There is also a relationship between skin elasticity and skin care practices in different cities, for example, women in Singapore had the highest skin elasticity compared to Vietnam and Indonesia (22, 43).

## 3 Differences in skin morphology across population groups

The skin is the largest organ of the human body, and in terms of macroscopic structure, it consists of three main layers: the epidermis, the dermis and the subcutaneous tissue. The characteristic structure varies among different populations.

### 3.1 Differences in epidermal structure

The epidermis is located in the outermost layer of the skin and is the barrier part of the skin, which is mainly composed of keratin-forming cells, melanocytes, etc., and is divided from the outside to the inside into the following layers: stratum corneum, stratum pellucidum, stratum granulosum, stratum spinosum, and stratum basale (57). The epidermis has the function of protecting the body from external physical, chemical and microbiological aggressions and is also involved in the regulation of water and electrolyte balance. The thickness of the epidermis varies depending on the body part, with the epidermis being thicker on the palms of the hands and soles of the feet and relatively thinner on the face.

Epidermal stratum corneum thickness varies, with studies generally reporting a thinner layer in individuals identified as East Asian and a thicker, more compact layer in those identified as Black. Using tape peel tests, it has been found that individuals identified as Black require more peels to disrupt the barrier, indicating greater barrier strength; individuals identified as White require an intermediate number; and those identified as East Asian require the fewest, suggesting weaker barrier strength (29, 58–60). However, with age, individuals identified as White experience a more rapid reduction in epidermal thickness and earlier signs of skin aging (61). In the granular layer, the size of granular layer nuclei increased with age in individuals identified as White, while it remained stable in those identified as East Asian (61).

In the basal layer, the size and distribution of melanosomes differed between individuals from different population groups, although the number of melanocytes was the same (60). Melanosomes in individuals identified as Black are larger and contain large amounts of melanin, whereas melanosomes in individuals identified as White skin are smaller, clustered and degrade more rapidly (60). Epidermal melanin in individuals identified as Black is predominantly true eumelanin, whereas White epidermis contains large amounts of pheomelanin, and the increase in true melanin makes individuals identified as Black more prone to hyperpigmentation disorders, such as post-inflammatory hyperpigmentation (60). All of these conditions contribute to the differences in skin color that occur across different population groups.

### 3.2 Dermal structural differences

The dermis is located beneath the epidermis and consists of dense connective tissue rich in collagen fibers and elastic fibers that give the skin its elasticity and toughness. It also contains accessory structures such as blood vessels, nerves, hair follicles, sweat and sebaceous glands, which are responsible for nutrient supply, sensory conduction, as well as secretion and excretion.

The dermis tends to be thicker and more compact in individuals with darker skin phototypes and thinner and less compact in those with lighter phototypes. The dermal papillary and reticular layers are more pronounced in individuals identified as White compared to those identified as Black, and the dermal papilla cells decrease more rapidly with age (61). Individuals identified as Black have been reported to have smaller bundles of collagen fibers, with proteoglycans tightly packed between the collagen and fibroblasts that are numerous, large and more active, expressing more of the elastin precursor tropoelastin and mature elastic fibers than skin of individuals identified as White, hence the propensity for keloid formation in individuals identified as Black descent (59, 60).

The dermal-epidermal junction (DEJ) in skin of individuals identified as Black shows a highly convoluted morphology with a high number and uniform distribution of papillary dermal and epidermal protrusions, whereas in skin of individuals identified as White the morphology of the DEJ is more varied, with a greater variation in the number and distribution of papillary protrusions, and a significant weakening of the structure with age, so that individuals identified as White are more prone to skin laxity and wrinkles (59, 61). In addition the length of the DEJ is approximately 2.4 times longer in skin of individuals identified as Black than in skin of individuals identified as White (59).

## 4 Differences in molecular phenotype across population groups

The molecular phenotype of the skin, including the composition and dynamics of lipids, proteins, metabolites and other molecules, plays a crucial role in the state of skin health. It determines the skin’s barrier function, moisturizing capacity, immune response, and anti-aging properties. Differences in the molecular phenotypes of skin from different populations may be responsible for differences in physiological indicators.

### 4.1 Genes

One study using DNA microarray analysis found that 181 epidermally expressed genes were up-regulated in the epidermis of individuals identified as Black, while 245 were up-regulated in the epidermis of individuals identified as White (62). This suggests that there are differences in the expression of genes in the skin of individuals from different population groups, and that these differences result in differences in molecular expression in the skin of individuals from different population groups, which in turn result in structural differences and are reflected by physiological indicators.

The most reported differences in gene expression between populations focus on differences in skin color (15). On the Solute Carrier Family 24 Member 5 (SLC24A5) gene, among individuals identified as White in the cited studies, the frequency of the 111A allele is close to 99%, almost reaching a fixed state, which has an important effect on light skin color; among populations identified as Black, the 111 T allele has a higher frequency, which is associated with darker skin color; however, the effect of this allele on skin color in populations identified as Indian is still controversial (63–66). The Agouti Signaling Protein (ASIP) gene in individuals identified as White is associated with blonde and red hair, whereas in individuals identified as Black, the ASIP gene has a lower frequency of variants, which may be associated with darker skin color (67–70). rs1800414*C 615R-derived genes on the Oculocutaneous Albinism 2 (OCA2) gene have a higher prevalence in East Asian populations, which may be associated with lighter skin color (71). Variants in the Major Facilitator Superfamily Domain Containing 12 (MFSD12) and Damage Specific DNA Binding Protein 1/Transmembrane Protein 138 (DDB1/TMEM138) regions are also associated with significant differences in skin color in Black populations (72). In addition, the region most strongly associated with skin color is the MFSD12 gene, specifically the rs2240751A/G (Y182H) variant, which is more common only in Native Americans and East Asians, as the MFSD12 region has undergone significant evolutionary selection in East Asians, and the frequency of the Y182H polymorphism correlates with the intensity of solar radiation and lighter skin color (73).

### 4.2 Proteins

Skin contains a variety of proteins that play a role in maintaining skin form and participating in skin function. Some of the common ones are collagen, elastin, and keratin. One study used quantitative proteomic analysis of skin from individuals identified as White and Black and found that 98 proteins were up-regulated in the skin model of individuals identified as White, while 34 proteins were up-regulated in the skin model of individuals identified as Black (62). These differences affect the state and function of the skin and are reflected by physiological indicators.

There are no significant differences in stratum corneum protein content among individuals from different population groups with healthy skin, but there were differences in certain types of proteins (74). As populations originating from regions with relatively cooler temperatures may have undergone distinct selective pressures, proteins associated with filaggrin polymerization proteolysis have been found to be more highly expressed in skin models derived from individuals identified as White, such as Bleomycin hydrolase (BH), Histidine Ammonia-Lyase (HAL), arginase, calpain-1 (C-1) and caspase 14 (62, 75–77). BH is involved in the final degradation of silk polymerization proteins and produces free amino acids that are important components of natural moisturizing factors (NMFs), which are expressed more in young and old skin and are thought to be a compensatory mechanism for dry skin, hence the higher TEWL in individuals identified as White as compared to those identified as Black (78). HAL converts histidine into trans-urocanic acid (tUCA), an important UV-absorbing pigment found in the stratum corneum, which absorbs UVB, thereby reducing skin damage and acting as a photoprotectant, thus influencing the relationship between skin pigmentation and vitamin D levels (79). Arginase is a key enzyme in the urea cycle, responsible for the metabolism of L-arginine to L-ornithine, which is involved in cellular detoxification, proliferation, and collagen synthesis (80, 81). Calpain-1 is a calcium-dependent cysteine protease involved in the degradation of filamentous proteins, which results in the production of natural moisturizing factors, which play an important role in the maintenance of the skin barrier function, and the formation and maturation of the stratum corneum. It plays an important role in the maintenance of the skin barrier function and in the formation and maturation of the stratum corneum, and its aberrant expression may lead to skin inflammation (82–84). The lower calpain-1 activity observed in individuals identified as Black may be due to the fact that this population has been reported to have a thicker and more compact skin structure and therefore may not require excessive humectants for regulation and protection. Caspase 14 is expressed in the stratum corneum and stratum granulosum of the skin, where it plays a role in maintaining the hydration status and barrier function of the skin, and has been implicated in inflammatory responses, The higher expression of caspase 14 in individuals identified as White skin may be due to the fact that the stratum corneum of individuals identified as White skin is more lax and requires more moisturizing factors to regulate it (85, 86).

Both TGF-*β*1 protein and TGF-β receptor 1 (TbR1) levels were significantly higher in the skin of individuals identified as Black than in those identified as White, TGF-β2 had a smaller difference between the two, and TGF-β receptor 3 (TbR3) protein levels were significantly lower in individuals identified as Black, and the expression of all three was closely related to elastin synthesis and protection against photo-aging so that aging is more pronounced in individuals identified as White (87).

Plasmin activity is reported to be lower in individuals identified as Black relative to individuals identified as White (88). Plasmin is involved in inflammatory responses in the skin, affects skin barrier function, promotes photoaging, and regulates skin cell differentiation and proliferation. Plasmin activates inflammatory factors and recruits inflammatory cells to exacerbate skin inflammation, and increases its activity in response to ultraviolet radiation, leading to collagen degradation and photoaging of the skin, and affecting the stratum corneum integrity and skin cell differentiation (89–91). Therefore, individuals identified as White tend to have a more fragile skin barrier and are more susceptible to photoaging compared to those identified as Black.

In cellular studies, significantly higher levels of monocyte chemotactic peptide-1 (MCP-1) protein and keratinocyte growth factor (KGF) were found in cell cultures from donors identified as Black than from those identified as White in cultures of papillary fibroblasts (59). The expression of MCP-1 is elevated in inflammatory dermatological disorders, such as psoriasis and eczema, and is involved in disease pathology and plays an important role in chronic inflammatory responses (92, 93). This may also be one of the reasons for the higher prevalence of atopic dermatitis among individuals identified as Black (94, 95). KGF maintains the normal thickness and function of the epidermis by promoting the proliferation and differentiation of keratinocytes and has some anti-inflammatory and photodamage-attenuating properties (96–98). These functions are responsible for the differences in skin structure between individuals identified as Black and those identified as White, and may also account for the greater susceptibility of individuals identified as White to photoaging.

### 4.3 Lipids

Skin lipids are an important part of the skin surface, produced by sebaceous glands, keratinocytes, and skin microorganisms, which play a role in maintaining the skin barrier function, moisturizing and nourishing the skin, protecting it from external stimuli, regulating inflammation, and maintaining the balance of the skin microbiota, thus revealing the microbiological situation in response to the state of the skin, and reflecting the process of skin aging and the mechanism of disease, which is very important in the field of dermatological sciences (5, 99–105).

It was concluded that the highest total lipid content of the stratum corneum was found in individuals identified as Black and the lowest in those identified as White, which is also in line with the findings of physiological indices determined in the previous section (39, 44). In the direction of specific subclasses, significant differences between ceramide subgroups were found between populations (46). The stratum corneum of individuals identified as Black has been reported to have a lower ceramide proportion and a weaker response of signaling pathways related to lipid/ceramide metabolism (44, 60, 62, 106). Ceramides play a role in the skin in maintaining the skin barrier, regulating skin hydration, ameliorating skin dryness, and repairing barrier damage related (100, 107–109). Skin of individuals identified as Black has higher moisture content, lower TEWL values, but lower ceramide content, which is contrary to our previous knowledge. This may be due to the fact that the function of ceramides is related to their type, structure, chain length, and saturation, or it may be due to the fact that skin of individuals with darker phototypes is structurally thicker and more compact, and therefore may not require as many ceramides for regulation and protection (109–112). This hypothesis is supported by studies showing that East Asian, who often have a thinner stratum corneum, tend to have a higher ceramide proportion (46, 60, 77).

In addition to common ceramides, pyrrolidone carboxylic acid (PCA) levels are significantly higher in individuals identified as Black than in those identified as White (88). PCA is one of the main components of NMF, which helps to maintain the skin barrier function, maintains the acidic environment of the skin, inhibits inflammatory factors to reduce the inflammatory reactions, absorbs and retains water, maintains the hydration status of the stratum corneum, and enhances the skin’s moisturizing capacity which may contribute to the higher hydration levels observed in individuals identified as Black. In addition, PCA stimulates fibroblast activity and promotes collagen synthesis (76, 113–115). This may contribute to the firmer skin phenotype observed in individuals identified as Black. Individuals identified as White, on the other hand, are often from populations originating in regions with cooler temperatures, and natural selection due to this cooler environment may have reduced PCA production in these populations (75).

In terms of wax ester content, wax ester content is significantly higher in individuals identified as Black than in those individuals identified as White and East Asian (34). Wax esters exhibited significant differences in the six fatty acids C14:0, C15:0, C16:1 (n-10), C17:1, C18:1 (n-9), and C18:2 iso (34). Differences in wax esters may contribute to the greater skin barrier function possessed by Black, whereas C15:0 and C17:1 may derive from metabolites of the skin microbiota.

Maturity of corneocytes originating, corneocyte surface area, Percentage of nucleated cells, and other metrics of individuals identified as Black and White, on the other hand, did not differ significantly (88).

### 4.4 Microecology

Skin microecology refers to the dynamic ecosystem of microbial communities (including bacteria, fungi, viruses, etc.) on the skin surface and their interactions with the host skin and the environment (116). These microorganisms protect against pathogen invasion, modulate immune responses, promote skin barrier function, and participate in metabolic processes (5, 6). A balanced skin microecology contributes to the prevention of infections, inflammation and skin diseases such as acne and eczema. In addition, it is closely associated with processes such as skin aging and wound healing (101, 117).

There is some similarity in the skin microbiology across populations with diverse geographic and ethnic backgrounds. For example, the microbiota in skin samples from individuals identified as Black (Cameroonians) and Japanese can be categorized into three phyla, Actinobacteria, Proteobacteria and Firmicutes; at the genus level, the most common sequences are Cutibacterium, Staphylococcus, Micrococcus, Corynebacterium, Kocuria (Cooke’s Bacteria), and to a lesser extent Janibacter and Streptococcus (118). The most abundant hand microbial communities in the United States and Tanzania were the thick-walled phylum Firmicutes and the anamorphic phylum Proteobacteria, respectively (119). In a study of individuals identified as White, American Black, continental Black, East Asian, Latin American, and South Asian in the United States, it was found that Actinobacteria and Thick-walled Bacteria phylums at the phylum level, and Staphylococcus and Corynebacterium at the genus level were found to be present in most of the groups (120).

There are also some differences in microorganisms across populations from different geographic regions (45, 120). Staphylococcus species were significantly higher than Corynebacterium species in Asians compared to Americans (121). Ochrobactrum was 40.0% and Propionibacterium was 30.4% Staphylococcus was 7.1% more prevalent in Korean skin (122). Cutibacterium had a significantly higher relative abundance in Japanese human skin (118). The highest proportions of Propionibacterium, Streptococcus and Staphylococcaceae were found in US samples (119, 121). The microbial community on the palms of Canadians consists mainly of Bacillus, Streptococcus and Propionibacterium (121). In microbiological studies of individuals identified as Black, soil-associated Rhodobacteraceae and Nocardiodaceae were the most dominant on the hands of Tanzanians, with the most abundant being the Proteobacteria (119). Micrococcus was dominant on skin samples from Cameroonians and had higher levels of microbial abundance than Japanese (118). Studies on Cameroonian cohorts did not report the high percentage of Staphylococcus spp. observed in some East Asian and North American samples, which may be related to the modulation of growth factors such as PCA in the previous article (76). Latin American populations are relatively rich in Proteobacteria and Acinetobacter (120).

Although the skin microbiomes of different populations share some commonalities in their basic composition, there are significant differences in the abundance and distribution of specific genera depending on factors such as population descriptor, lifestyle, and environment, and these differences may have an impact on skin health and disease development (120, 123). These differences may be due to the fact that the skin environment is more nutrient-poor, and for skin surface microorganisms, skin lipids are the best and most important source of carbon and nitrogen, which are the drivers of the species and distribution of skin surface microorganisms. Therefore, the joint study of skin lipids and microorganisms can help to further explain the skin microecological differences and molecular mechanisms among individuals from different population groups.

## 5 Conclusion

Skin phenotypes—including transepidermal water loss, moisture content, sebum production, elasticity, and pH—differ significantly across populations with varying skin phototypes as reported in the literature. These differences reflect the complex interplay of genetic adaptation to diverse environmental conditions, as well as ongoing gene–environment interactions. For example, individuals with darker skin phototypes tend to have firmer skin but lower ceramide proportions, whereas individuals with East Asian ancestry tend to have lower hydration and a thinner stratum corneum. In addition, environmental factors also have an important influence within the same population group, e.g., the ability to dissipate water and the moisture content of the skin varies among individuals identified with the same population descriptor in different regions, depending on environmental conditions such as UV exposure, temperature and humidity.

In addition to the screening effect of natural selection on skin function, differences in skin physiological indices are directly attributable to differences in skin structure and molecular phenotypes across populations with varying skin phototypes. Although studies have been conducted to reveal some of these differences and their causes, there are still many questions that need to be further explored. For example, what are the similarities and differences in the interactions of different types of skin barriers (physical, chemical, immune, and microbial) across populations with varying skin phototypes; the differences in different protein or lipid isoforms across populations and their specific impact on skin barrier function; and the interactions between microorganisms and skin structures and molecules, and their impact on the overall function of the skin.

These explorations of skin structure, mechanism, and function must take full account of population-level variation and the complex interplay of genetic, environmental, and social factors that shape skin phenotypes. So as to help diagnose and treat skin diseases more accurately and provide a more scientific theoretical basis for the research, development and regulation of related drugs and cosmetics.

## 6 Limitation

Several limitations inherent to this review should be acknowledged. First, the racial and ethnic categories used in the cited primary studies are social and political constructs rather than fixed biological entities, and their definition and self-identification vary across populations and over time. Second, there is considerable methodological heterogeneity among the included studies in how these population descriptors were defined, recruited, and classified, which complicates direct cross-study comparisons. Third, the original publications rarely provided data on key social determinants of health (e.g., socioeconomic status, education, lifestyle, or environmental exposures), which may confound or modify the observed skin phenotypes. Finally, despite the grouping by broad categories, the data reviewed here consistently demonstrate substantial within-group variability, underscoring that these aggregate labels cannot adequately capture the full spectrum of individual skin characteristics. These limitations should be considered when interpreting the findings, and they highlight the need for future research to adopt standardized, phenotype-focused frameworks that explicitly account for both biological and social dimensions of human diversity.
